# Linking anthropogenic resources to wildlife–pathogen dynamics: a review and meta-analysis

**DOI:** 10.1111/ele.12428

**Published:** 2015-03-21

**Authors:** Daniel J Becker, Daniel G Streicker, Sonia Altizer

**Affiliations:** 1 Odum School of Ecology, University of GeorgiaAthens, GA, USA; 2 Institute of Biodiversity, Animal Health and Comparative Medicine, University of GlasgowGlasgow, G12 8QQ, UK; 3 MRC-University of Glasgow Centre for Virus ResearchGlasgow, G61 1QH, UK

**Keywords:** Aggregation, agriculture, foraging ecology, host–parasite interactions, immune defence, infectious disease ecology, mathematical models, supplemental feeding, urbanisation

## Abstract

Urbanisation and agriculture cause declines for many wildlife, but some species benefit from novel resources, especially food, provided in human-dominated habitats. Resulting shifts in wildlife ecology can alter infectious disease dynamics and create opportunities for cross-species transmission, yet predicting host–pathogen responses to resource provisioning is challenging. Factors enhancing transmission, such as increased aggregation, could be offset by better host immunity due to improved nutrition. Here, we conduct a review and meta-analysis to show that food provisioning results in highly heterogeneous infection outcomes that depend on pathogen type and anthropogenic food source. We also find empirical support for behavioural and immune mechanisms through which human-provided resources alter host exposure and tolerance to pathogens. A review of recent theoretical models of resource provisioning and infection dynamics shows that changes in host contact rates and immunity produce strong non-linear responses in pathogen invasion and prevalence. By integrating results of our meta-analysis back into a theoretical framework, we find provisioning amplifies pathogen invasion under increased host aggregation and tolerance, but reduces transmission if provisioned food decreases dietary exposure to parasites. These results carry implications for wildlife disease management and highlight areas for future work, such as how resource shifts might affect virulence evolution.

## Introduction

Human activities and changes to the landscape can dramatically alter the types, abundance, and distribution of resources available to wildlife. These changes can affect nesting structures, shelter, and water but are particularly apparent for food resources. Urbanisation, agricultural intensification, and overfishing have depleted food abundance for many wildlife through habitat degradation and reduction in prey stocks (Lotze *et al*. 2006; Fischer & Lindenmayer 2007). Many species decline in response to such activities, but some generalists thrive in human-dominated habitats by capitalising on novel food resources (McKinney 2006; Sih *et al*. 2011).

Human provisioning of wildlife with food is geographically widespread, occurs at local and landscape scales and can be intentional or accidental (Oro *et al*. 2013). Bird feeders, supplemental feeding stations and wildlife tourism are examples of intentional provisioning (Cross *et al*. 2007; Newsome & Rodger 2008; Robb *et al*. 2008), whereas accidental food can be provided through agriculture, household waste and landfills (Fedriani *et al*. 2001; Gauthier *et al*. 2005; Ciach & Kruszyk 2010). The high abundance and predictability of these resources across space and time can make them accessible components of wildlife diets, potentially resulting in populations that are larger, more aggregated and better-fed (Boutin 1990; Oro *et al*. 2013). Subsidised wildlife populations can, in turn, influence ecological processes ranging from trophic cascades to alternative stable states (Jefferies *et al*. 2004; Newsome *et al*. 2015).

A growing number of studies indicate that anthropogenic resources can alter host–pathogen interactions, leading to either increased or decreased infection risk for wildlife and humans depending on the nature of provisioning and the particular host–pathogen interaction (Table1). Heterogeneity in infection outcomes observed to date underscores the need for conceptual frameworks to reconcile these divergent consequences. This is especially important given that provisioning frequently brings different host species into contact and could facilitate host shifts and novel pathogen emergence, with consequences for wildlife conservation and human health (Bradley & Altizer 2007). For example, bird feeders have been implicated in the spread of several songbird pathogens, including mycoplasmal conjunctivitis and a virulent strain of trichomoniasis, in part owing to transmission opportunities created by the close proximity and large aggregations of birds around human-provided food sources (Table1; Dhondt *et al*. 2005; Lawson *et al*. 2012). In Malaysia, the planting of fruit trees near pigsties is known to attract fruit bats to forage nearby, providing opportunities for the cross-species transmission of Nipah virus from bats to pigs and leading to human exposures (Table1; Field *et al*. 2001).

**Table 1:** Examples of host–pathogen interactions that respond to provisioning, including the anthropogenic resources provided, pathogens affected and observed impacts on the host

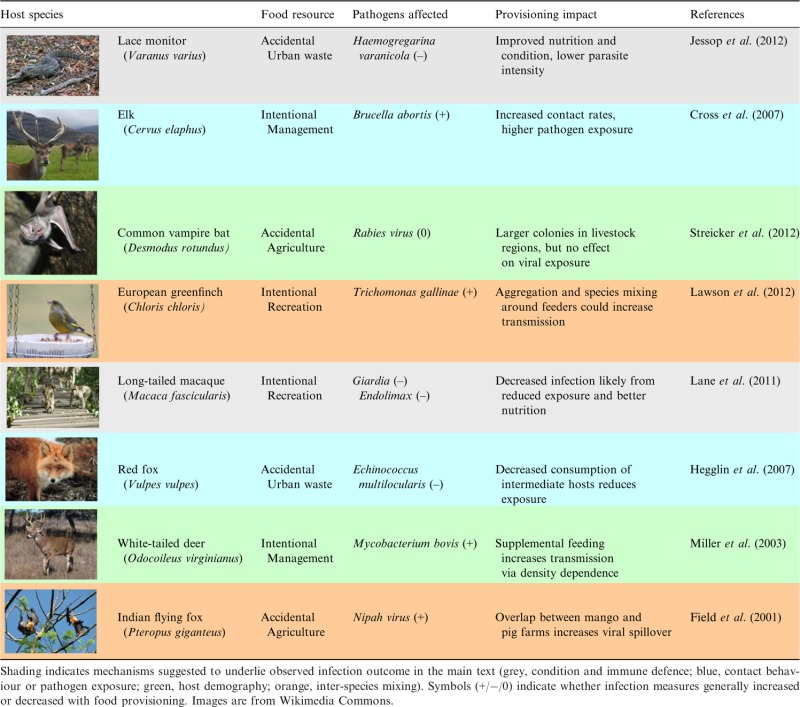

**Figure 1:**
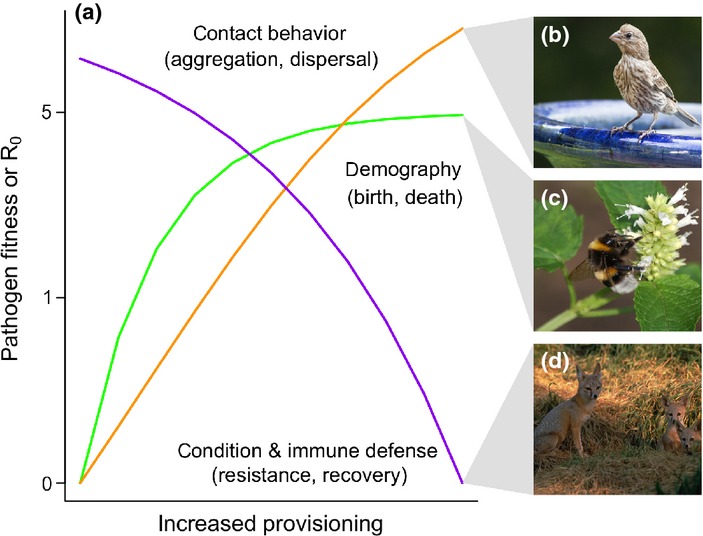
Predicted relationships between provisioning and *R*_0_ (where *R*_0_ = 1 is the pathogen invasion threshold). Aggregation around resources could increase host contact rates and infectious stage build-up in the environment (a; orange), an effect illustrated by increased flocking of house finches at bird feeders and associated increases in conjunctivitis prevalence (b; Altizer *et al*. 2004). Provisioning can also improve host vital rates and increase host population sizes (a; green), which was suggested to explain higher pathogen prevalence among bumblebees in urban versus rural gardens (c; Goulson *et al*. 2012). Positive effects of provisioning on *R*_0_ could be countered by improved host condition and immune defence (a; purple). Such an effect is suggested by kit foxes showing lower nutritional stress, higher body condition, and improved immune function in urban areas where food and water was more plentiful (d; Cypher & Frost 1999). Images are provided by Wikimedia Commons.

Here, we provide a conceptual framework for understanding how provisioning affects infection dynamics in wildlife and consider the practical implications for pathogen emergence and control. We start by reviewing empirical support for three mechanisms through which provisioning can affect host–pathogen interactions by altering (1) host immune defences, (2) host contact and movement behaviours and (3) host demography (Fig.1). These mechanisms can operate simultaneously and might have divergent effects on population-level disease outcomes. We next conduct a meta-analysis of empirical studies to characterise the range of outcomes observed in response to provisioning and assess the importance of host, pathogen and environmental factors in determining whether infections increase or decrease in response to anthropogenic resources. Our analyses provide support for behavioural and immunological processes by which exposure, resistance and tolerance are altered by provisioning. These analyses also identify pathogen type and food source as determinants of infection outcomes. To synthesise these findings, we review theoretical models examining the effect of provisioning on pathogen dynamics and integrate our meta-analysis results back into a mechanistic and predictive framework using the basic reproductive number *R*_0_, a threshold quantity determining whether a pathogen can invade a host population, as a measure of pathogen fitness (Anderson & May 1991). We conclude by highlighting the management implications of our analyses and suggest avenues for future research on how wildlife–pathogen interactions respond to anthropogenic resources.

## Mechanistic links between provisioning and pathogen infection

### Feeding a fever: how resources alter immune defence

A host's ability to mount defences and recover from infection depends on its nutritional state, which is influenced by both food quantity and quality (Nelson 2002). Studies in humans, mice and poultry show that energy and protein deficiencies can weaken immune cell function and complement proteins (Chandra 1999; Klasing 2007). Moreover, nutrient deficiencies (zinc; iron; beta-carotene; vitamins B6, B12, C, D and E; and folic acid) can impair immune defence, especially in young and old individuals (Chandra 2004; Cunningham-Rundles *et al*. 2005). Malnourished wildlife can become immunosuppressed, which can increase pathogen replication and lead to higher host morbidity and mortality (Coop & Kyriazakis 2001; Ezenwa 2004). Therefore, by providing reliable food resources, provisioning could boost wildlife body condition and increase immune defences, and could also allow wildlife to spend less time foraging and budget more time towards behavioural defences such as grooming. This, in turn, could reduce pathogen fitness by decreasing individual susceptibility to pathogens and shortening the time to recovery following infection (Fig.1).

In support of this idea, work on kit foxes in California showed animals in residential areas to be in better condition compared to animals occupying a reserve, which weighed less and showed signs of dehydration and tissue catabolism (Fig.1d; Cypher & Frost 1999). Although exposure to three canine viruses was similar between the two groups, haematology suggested better immune status in urban foxes, likely owing to improved access to water and food and in turn reduced risk of starvation. Other studies of lace monitors foraging on human refuse found that resource-mediated increases in body condition were associated with lower intensity of blood parasites compared to unprovisioned animals (Table1; Jessop *et al*. 2012).

Under some conditions, supplemental resources could have the opposite effect of increasing host susceptibility to infection, which should increase rather than reduce *R*_0_. Abundant but poor-quality anthropogenic food sources that are low in protein or high in fat could impair immune function, especially antibody-mediated defences (van Heugten *et al*. 1996; Maggini *et al*. 2007). Although direct support for dietary shifts causing increased disease susceptibility in wildlife is rare, several case studies suggest this could occur. For example, supplemental feeding of rock iguanas by tourists in the Bahamas with carbohydrate-rich foods such as cereals and grapes was associated with altered nutritional status and increased hookworm burdens (Knapp *et al*. 2013). Similarly, southern stingrays fed by tourist boat operators in the Cayman Islands experienced impaired physiology resulting from poor nutrition and stress arising from crowding (Semeniuk *et al*. 2009). In addition, some forms of provisioning could enhance pathogen transmission by improving host tolerance to infection, thus allowing heavily infected animals to better survive and shed infectious stages (Råberg *et al*. 2009; Vale *et al*. 2013).

### Stay awhile and eat: resource-driven changes in host aggregation and dispersal

By providing concentrated and reliable resources, provisioning can reduce host foraging ranges, promote aggregation and might favour more sedentary behaviour as animals move less in search of food (Boutin 1990). Such changes in response to greater resources have been observed in urban feral cats, which show more localised foraging with greater territory overlap around supplemental feeding stations compared to rural cats (Schmidt *et al*. 2007). Higher aggregation and local host density could increase host contact rates, which should increase *R*_0_ (McCallum *et al*. 2001; Fig.1). In support of this idea, wild raccoons experimentally provisioned with concentrated food resources had greater contact rates, resulting in higher prevalence of endoparasite infections (Wright & Gompper 2005). In addition, elevated contact rates from flocking at bird feeders were suggested to cause greater spread of mycoplasma conjunctivitis in house finches (Fig.1b; Altizer *et al*. 2004). Importantly, a positive response of pathogens to host aggregation requires that contact rates and pathogen transmission scale positively with local host density (Lloyd-Smith *et al*. 2005). As one example, studies of vampire bats in Latin America suggest that the growing availability of blood meals from livestock rearing has facilitated range expansions and population growth of this host, which serves as the key reservoir for rabies virus (Lee *et al*. 2012). However, despite a weak positive relationship between livestock density and bat colony size, rabies virus exposure was not associated with the latter, indicating that contact rates between susceptible and infected bats might not increase with host density (Table1; Streicker *et al*. 2012).

Stable food sources might decrease host foraging movements and could encourage migratory or nomadic species to form sedentary populations (Altizer *et al*. 2011). For example, Spanish white storks in recent years have abandoned long-distance migration to Africa and instead now overwinter on urban landfills close to their breeding range (Ciach & Kruszyk 2010). Reduced host movement could increase pathogen transmission by allowing year-round exposure to pathogens that accumulate in the environment (Altizer *et al*. 2011; Hall *et al*. 2014). Importantly, sedentary populations could also lose connectivity with other groups, as has been suggested by work on urbanised flying foxes in Australia, leading to local viral extinction over short timescales and setting the stage for larger outbreaks following pathogen reintroduction (Plowright *et al*. 2011).

Some host behavioural responses to provisioning could decrease infection risk, especially for parasites commonly encountered in the course of wildlife foraging activity, such as those with complex life cycles involving intermediate hosts. Work on Balinese long-tailed macaques suggested that increased feeding on tourist-provided food decreased the prevalence and intensity of several gastrointestinal protozoa (Table1; Lane *et al*. 2011), possibly because provisioned habitats and food were relatively free of infectious stages found in natural environments. Similarly, provisioning decreased the prevalence of helminths recovered from subsidised raccoons and red foxes, possibly because hosts switched diets away from feeding on naturally infected intermediate hosts (Table1; Hegglin *et al*. 2007; Monello & Gompper 2011).

### Food for the masses: how resources influence wildlife demography

Pathogen invasion and persistence rely on the supply of new susceptible hosts through births or immigration. Supplemental feeding has been shown to increase fecundity or shorten the time to first reproduction across a range of animal taxa (Boutin 1990; Krebs *et al*. 1995; Nagy & Holmes 2005). Since offspring are typically born immunologically naïve (or become so after waning of maternal antibodies), heightened reproduction can increase the number of susceptible individuals and thereby elevating *R*_0_ (Fig.1). In addition, provisioning can reduce juvenile mortality rates by reducing starvation and improving overall condition, further contributing to the pool of susceptible hosts (Ozoga & Verme 1982).

If novel resources increase local carrying capacities for wildlife, this could favour pathogen transmission by two well-known processes: the critical community size (a threshold population size at which stochastic extinction of pathogens becomes unlikely) and density-dependent transmission (in which pathogen prevalence scales positively with host density; McCallum *et al*. 2001). Evidence to date for pathogen responses to provisioning-altered host demography is primarily indirect. For example, supplemental feeding of white-tailed deer and red deer elevates host densities, which has been suggested to increase the prevalence of bovine tuberculosis (Table1; Miller *et al*. 2003; Vicente *et al*. 2007). Similarly, urban gardens in Scotland had greater bumblebee densities and higher prevalence of multiple pathogens (Fig.1c; Goulson *et al*. 2012), although direct links between population size and infection were not examined.

Although effects of provisioning on demographic processes are generally expected to increase transmission, complex patterns could arise for immunising pathogens. If provisioning prolongs the survival of previously exposed immune individuals more than it stimulates fecundity, this could increase herd immunity and reduce pathogen transmission. Thus, understanding precisely which demographic processes respond to anthropogenic resources and how this affects the age, sex and immunological structure of populations is critical to anticipate the consequences of provisioning for host–pathogen dynamics.

## Meta-analysis of provisioning effects on infection outcomes

The examples and mechanisms noted above suggest that resource provisioning can generate wide variation in pathogen fitness. To better characterise the range of infection outcomes and to identify key predictors of this variation, we conducted a meta-analysis of empirical studies of microparasites (viruses, bacteria, protozoa, fungi) and macroparasites (helminths and ectoparasites). We focused on studies that recorded either pathogen prevalence (proportion of individuals infected), seroprevalence (proportion displaying a pathogen-specific immune response), or intensity of infection (average number of parasites per infected host) in provisioned and unprovisioned wildlife populations. Our specific goals were to (1) characterise the breadth of studies in the provisioning–disease literature; (2) identify the range and average responses of infection; (3) identify host, parasite and environmental factors that best explain variation in observed infection outcomes; and (4) test empirical support for our proposed mechanisms of immunological, behavioural, and demographic changes.

### Literature survey and statistical approach

Scholarly articles were identified through Web of Science, Google Scholar, CAB Abstracts, and PubMed searches using strings of terms relevant to anthropogenic resources, wildlife ecology, and pathogen transmission. Our systematic search identified 144 studies meeting criteria for inclusion, of which 23 provided infection measures (prevalence, intensity, or seroprevalence). From each study, we recorded the relationship between provisioning and infection measures (effect size and directionality) along with the source and intention of provisioning, host and pathogen type, and transmission mode of the pathogen. Because many studies reported data for multiple pathogens or hosts, our data set included 132 records, where each record consisted of a particular host–pathogen combination. Further details on search procedures, criteria for study inclusion, categorical assignments, descriptive analyses, and tests of publication bias are provided in the Supporting Information.

To test support for mechanisms described above, we recorded whether studies quantified host condition or immune defence, contact behaviour, or demography as well as how these measures covaried with provisioning. For the first mechanism, we considered studies that included body condition indices (e.g. mass∼length residuals or subjective scoring) or quantified immune function (e.g. humoral or cellular components). Behavioural measures included group size, time animals spent foraging, dietary complexity, and contact rates. Demographic variables included host density and population size (as birth and death rates were generally not reported). Of the 23 studies, 52% (*n *= 12) quantified host condition or immune defence, 43% (*n *= 10) quantified behavioural changes, and 26% (*n *= 6) quantified demography (Fig. S3).

We collected standardised effect sizes from reported test statistics (e.g. *r*^2^, odds ratios, *χ*^2^) and sample sizes for each provisioning–infection outcome. When authors did not report test statistics, we derived effect sizes by simplifying data to a contingency table. If comparisons were made between several provisioned and unprovisioned groups, samples were pooled to calculate chi-squared statistics with Yates correction for prevalence or Hedges *g* for intensity (Rosenthal & DiMatteo 2001). If comparisons were made between different categories of provisioning, measures were compared between the most extreme levels (Cooper *et al*. 2009). We converted effect sizes into the correlation-based *r* (Rosenthal & DiMatteo 2001; Bonett 2007) and assigned a negative value to cases where provisioning significantly reduced infection. Directional *r* effect sizes were transformed using Fisher's *Z* to stabilise variance (Fisher 1921).

Our analysis used random-effects models (REM) to infer the average effect of provisioning on infection. Next, we used mixed-effects models (MEM) to explain variation in infection according to pathogen type, transmission mode, host taxonomy, and provisioning type and source. Model simplification used backward removal of the least significant variable using Wald-type chi-squared tests followed by nested likelihood ratio tests (Van Houwelingen *et al*. 2002). We calculated contrasts for our best-fit MEM to test if coefficients differed significantly from zero after adjusting for the potentially inflated false-discovery rate associated with multiple comparisons, using the Benjamini and Hochberg correction and the *multcomp* package in R (Benjamini & Hochberg 1995; Bretz *et al*. 2010; R Core Team 2013). Finally, we used MEM to test support for effects of resource-altered host condition, behaviour, and demography on infection in each data subset reporting these variables. We used the R package *metafor* for *r*-to-*Z* effect size conversions and REM and MEM analyses (Viechtbauer 2010; R Core Team 2013).

### Drivers of infection outcomes following provisioning

**Figure 2:**
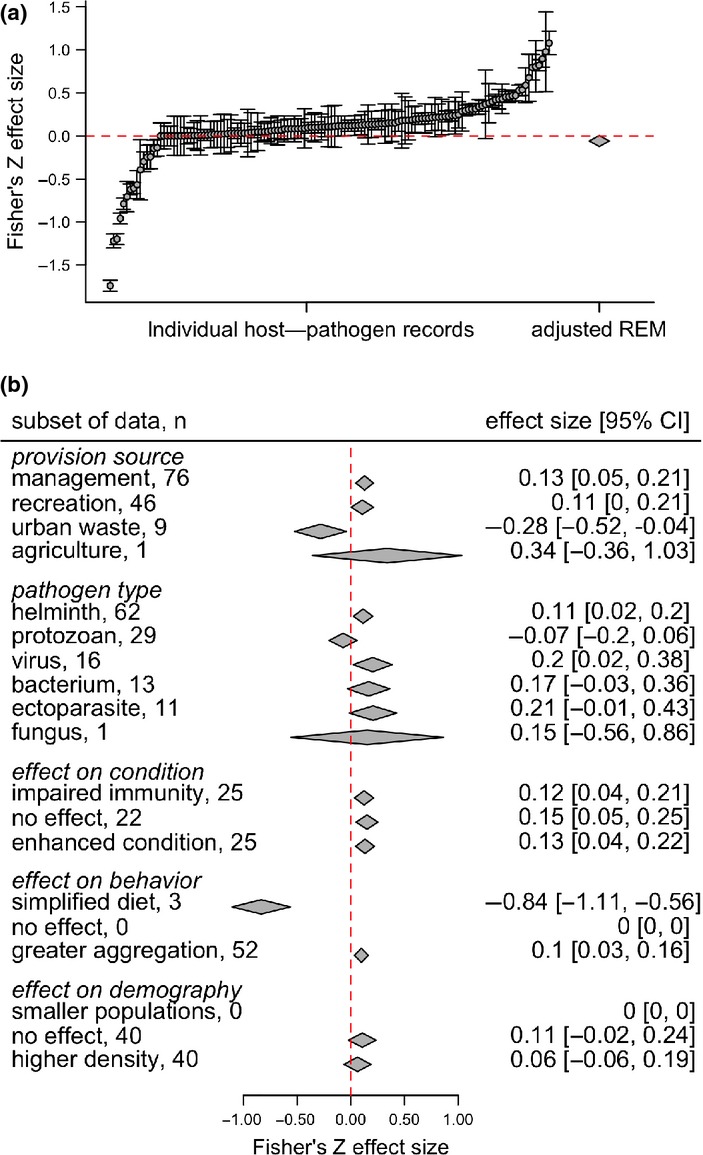
Distribution of effect sizes for observed relationships between provisioning and infection outcomes (points ± 95% confidence intervals) alongside the mean effect size estimate (diamond) from the bias-corrected REM (a). Each point is a particular host–pathogen interaction. Points above the horizontal line demonstrate cases where provisioning increased infection prevalence, intensity or seroprevalence; points below the horizontal line demonstrate reduced infection outcomes. (b) Estimated mean effect size of predictors on infection outcomes, denoted through diamonds alongside 95% confidence intervals. Sample size (*n*) refers to the number of host–pathogen interactions corresponding to each level. Positive effect sizes indicate increases in infection outcomes (measures of prevalence, seroprevalence and intensity are pooled).

Our meta-analysis demonstrated that provisioning is associated with a wide range of infection outcomes in wildlife (Fig.2a). Of the 132 wildlife–pathogen interactions identified, most showed no relationship between provisioning and infection measures (65%, *n* = 86), with 24% (*n* = 31) identifying positive and 11% (*n* = 15) identifying negative effects of anthropogenic resources. After adjusting for missing data due to suppression of extreme or non-significant results (Fig. S4), there was significant heterogeneity in infection outcomes (τ^2^ = 0.18; *Q* = 16902, d.f. = 176, *P *< 0.001) but no net directional effect of provisioning in the REM (*z* = −1.79, *P* = 0.07; Fig.2a).

**Figure 3:**
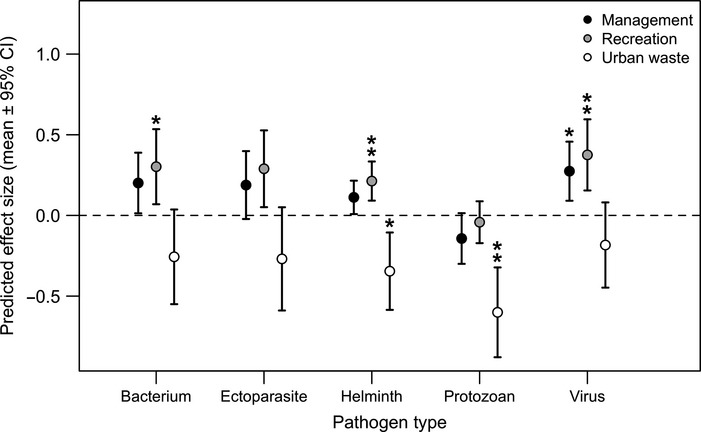
Visualisation of the MEM explaining the most variation in infection outcomes from the meta-analysis. Data points represent the predicted outcome of provisioning for each combination of food source (see legend) and pathogen type, where the horizontal line represents no influence of supplemental feeding on infection. Asterisks represent means significantly different from zero after adjusting for multiple comparisons (*P < 0.05, **P < 0.01). Effects based on agricultural food and fungal pathogens are not shown owing to limited data.

MEM analysis of individual covariates demonstrated that pathogen type, transmission mode, provisioning type and source, and, host taxonomy explained significant variation in infection outcomes (Table S2 and Fig. S5). Stepwise model selection and AIC further identified pathogen type and provisioning source as the strongest predictors (LRT = 25.54, d.f. = 2, *P *= 0.001; Table2). Univariate MEMs of these covariates showed that hosts provisioned intentionally by wildlife management and recreational resources had higher infection measures, whereas hosts foraging on unintentionally provided sources in urban areas experienced reduced infection (Fig.2b). In addition, infection measures for helminths and viruses generally increased with provisioning, whereas ectoparasites, bacteria, and protozoa showed no general response (Fig.2b). Our additive MEM integrating food source and pathogen type predicted infection with bacteria, helminths, and viruses to be significantly increased in recreational feeding areas (bacterium: *z* = 2.54, *P *= 0.04; helminth: *z* = 3.44, *P* = 0.01; virus: *z* = 3.34, *P* = 0.02), whereas infection with helminths and protozoa was predicted to be significantly reduced in hosts feeding on urban waste (helminth: *z* = −2.82, *P* = 0.02; protozoan: *z* = –4.23, *P* = 0.001; Fig.3). Low sample sizes for agricultural sources of provisioning and fungi prevented detailed analysis of their relative effects.

**Table 2:** Rank of competing MEM of provisioning effects on infection, including the *R*^*2*^ derived from likelihood ratio tests against the base REM

MEM	weight	ΔAIC	*R*^*2*^
Pathogen + source	0.50	0.00	17.85
Pathogen + source + host	0.40	0.44	20.77
Pathogen + source + host + transmission	0.04	5.04	21.62
Source	0.03	5.36	7.58
Pathogen + source + host + transmission + type	0.02	7.01	21.64
Type	0.01	8.12	2.51
Pathogen	0.00	10.48	6.77
Host	0.00	11.86	2.59
Transmission	0.00	14.55	0.61

REM, random-effects models; MEM, mixed-effects models.

We found mixed support for effects of resource-altered host immunity, behaviour, and demography on infection outcomes (Fig.2b). Studies quantifying host condition or immune function showed roughly even evidence for positive, negative, and no responses of these variables to provisioning (Fig. S3). MEM analysis indicated that responses of condition to provisioning predicted changes in infection (*Q* = 24.8, d.f. = 3, *P* < 0.001). Somewhat surprisingly, both greater (*μ* = 0.13, *z *= 2.97, *P *= 0.003) and poorer (*μ* = 0.12, *z* = 2.75, *P* = 0.006) host condition in provisioned wildlife were associated with greater infection measures (Fig.2b). Behavioural responses to provisioning also explained variation in infection (*Q* = 45.3, d.f. = 2, *P* < 0.001). Studies quantifying host behaviour primarily found contact and aggregation to increase with provisioning (Fig. S3), which was associated with greater infection measures (*μ* = 0.098, *z* = 3.02, *P* = 0.03). A subset of studies also found dietary diversity to decrease in provisioned populations, which was associated with lower infection measures (Fig.2b; *μ* = –0.8435, *z* = –6.01, *P* < 0.001). This pattern could arise if provisioning reduces parasite exposure through decreased consumption of intermediate hosts or infectious stages in natural food. Lastly, demographic variables (abundance, density) showed either no effect or positive responses to provisioning (Fig. S3), but these differences did not predict infection outcomes (*Q* = 3.58, d.f. = 2, *P* = 0.16; Fig.2b).

Our meta-analysis demonstrates that wildlife–pathogen responses to provisioning vary widely, with pathogen type and food source explaining the greatest variation in infection. Some pathogens that increased in response to provisioning, such as *Mycobacterium bovis* in deer and *herpesvirus* in raptors*,* are spread through close contact, while others such as *Cryptosporidium* in possums are transmitted through environmental infectious stages. Both transmission routes could be favoured if provisioning increases host aggregation and encourages sedentary behaviour, increasing exposure to infected conspecifics and to pathogens shed into the environment. The source of provisioning also predicted variation in infection outcomes, with intentionally managed and recreational resources generally increasing infection. For example, feeding stations to manage elk in the greater Yellowstone area during the winter months attract high densities of hosts, support sedentary behaviour, and allow for the build-up of environmentally transmitted parasites, and in turn increase exposure to bacterial pathogens and helminths (Table1; Cross *et al*. 2007; Hines *et al*. 2007). In another study, feeder station density was associated with greater nematode prevalence and intensity in wild boar (Navarro-Gonzalez *et al*. 2013). Accordingly, our best model predicted infection with such pathogens to be highest in hosts foraging at managed and recreational resources (Fig.3), lending support to provisioning amplifying transmission by creating hubs of high host contact and pathogen shedding in supplemented feeding environments.

Another mechanism to explain increased helminth transmission in provisioned habitats could be that well-fed hosts constitute a better reproductive environment for macroparasites (Seppälä *et al*. 2008), as supported through our finding that resource-improved condition predicts greater infection. Yet our analysis also found that provisioning can reduce body condition and immune function in some wildlife species, which was similarly associated with increased infection. Two non-exclusive mechanisms could underlie this pattern. First, some provisioned resources might be of low quality and lack nutrition, especially protein, needed for mounting immune defences (van Heugten *et al*. 1996; Coop & Kyriazakis 2001). Food provided to wildlife with good intentions could also contain contaminants that hamper immune defence. For example, one study in our analysis found supplemental food used to improve breeding success of imperial eagles contained pharmaceuticals that depressed immune function and elevated infection by multiple pathogens (Blanco *et al*. 2011). Second, crowding and high intraspecific competition around novel resources could function as a stressor that impairs host condition (Shochat 2004). One study of tourism in our analysis suggested this process, as provisioned stingrays intensively competed for food and in turn showed lower condition, higher injury rates, and increased ectoparasites burdens (Semeniuk & Rothley 2008). From a broader perspective, these results demonstrate negative fitness consequences of anthropogenic resources, suggesting that some provisioned habitats function as ecological traps for wildlife (Battin 2004).

Altogether, our findings provide support for several processes by which provisioning elevates host exposure and susceptibility to pathogens. However, our analyses also support pathways by which pathogen transmission is lowered in response to novel resources. Our best-supported model showed that hosts foraging on resources unintentionally provided in urban habitats experienced reduced infection with protozoa and helminths (Fig.3). This result may be driven by dependence on trophic transmission, for which shifts towards easily accessible anthropogenic food could reduce the consumption of natural intermediate hosts. In one study included in our analysis, reduced dietary breadth of ring-billed gulls foraging in urban areas was associated with lower helminth burdens, as birds fed more on urban waste and less on naturally infected intermediate hosts such as snails and crustaceans (Aponte *et al*. 2014).

Surprisingly, despite support for increases in host population size and density following provisioning, we found no effects of these demographic responses on infection outcomes. The failure of infection outcomes to scale with demographic patterns might reflect a dominance of frequency-dependent rather than density-dependent transmission in the studies analysed (McCallum *et al*. 2001). Alternatively, the particular demographic process (birth or survival) that is affected by provisioning might have a stronger impact than change in population size. In particular, if provisioning increases survival more than reproduction, this could decrease transmission through a build-up of herd immunity. This highlights the importance of measuring not just population size, but also the underlying demographic process generating larger population sizes in provisioned populations.

## Integrating resources into epidemiological models

Our review and meta-analysis suggest multiple processes through which provisioning can alter infectious disease dynamics. Because these mechanisms can act simultaneously and with potentially opposing directional effects, modelling approaches are critical for predicting the overall effect of provisioning on pathogen invasion and spread. Below, we review several recent studies that used empirically informed mechanistic models to better understand how host resources affect pathogen dynamics. We then integrate the best-supported relationships from our meta-analysis back into a mechanistic framework to gain a deeper understanding of processes underlying the observed variation in infection outcomes.

### Review of resource-dependent modelling approaches

Mathematical models that examine food provisioning and infectious disease dynamics include both system-specific and general theoretical approaches. Motivated by field observations showing that prevalence of fungal pathogens of *Daphnia* increased when lake resources were poor and declined when resources improved, one study integrated experimental resource manipulation with a mechanistic modelling approach (Hall *et al*. 2009). Experiments showed positive relationships between resource quality and both host fecundity and fungal spore production, which likely favoured transmission; however, greater resources also lowered host susceptibility to infection, slowing down transmission. When these empirical relationships were integrated into a compartmental model tracking the density of susceptible and infected hosts as well as free-living spores, simulations showed that *R*_0_ was maximised at intermediate resources and declined at both high and low resource levels.

**Figure 4:**
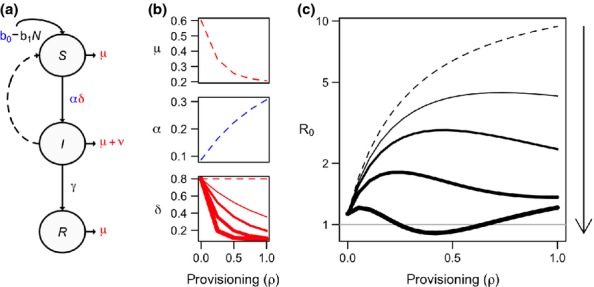
General modelling framework for how provisioning affects infectious disease dynamics of a microparasite (Box 1). In this compartmental framework (a–b), provisioning causes key parameters to increase (shown in blue) or decrease (shown in red). Varying the response of immune parameters to provisioning generates a range of outcomes on *R*_0_ (c). An increasingly saturating effect of provisioning is shown through line width (dashed indicates no effect on immunity), and this approach can generate outcomes ranging from amplifying prevalence to driving *R*_0_ below the invasion threshold (grey line). Figure is adapted from Becker & Hall (2014), and further model details and parameter definitions are provided in Box 1.

A more general modelling approach by Becker & Hall (2014) examined how resource-modified host demography, contact behaviour, and immune defence alter the transmission of close-contact microparasites (Box 1). By coupling functional responses of parameters including host birth and death rates, infection probability, tolerance of infection, and contact rates to provisioning, this model generated a range of pathogen invasion outcomes (Fig.4). In particular, host immune defence emerged as critical to predicting the net effect of provisioning on *R*_0_. When provisioning had minimal effects on host immunity, the positive effects of provisioning on host density and contact rates resulted in higher pathogen invasion. Yet when immune defence increased with provisioning in a saturating response, pathogen extinction occurred at intermediate resource levels and invasion was only possible at low and high resources (Fig.4), a pattern opposite of the modelling outcomes of Hall *et al*. (2009). In the latter model, low immunity at low resource levels allows pathogen invasion despite relatively low contact rates. At intermediate resources, high resistance to pathogens leads to low prevalence or pathogen extinction. At even greater resource levels, the continued increase in host contact rates overcomes host resistance and allows the pathogen to reach high prevalence (Fig.4). By demonstrating that *R*_0_ changes along a gradient of provisioning and by showing how this depends on underlying individual-level effects of resources, this approach provides a useful reference point for understanding the divergent patterns in disease outcomes observed in prior empirical work.

**Box 1 A compartmental model of microparasite dynamics in response to provisioning**

Because resource provisioning simultaneously affects individual- and population-level processes that can interact in opposing ways, mechanistic models can help resolve the net outcome for host–pathogen dynamics. In a modelling framework describing the effects of provisioning on microparasite systems outlined by Becker & Hall (2014), hosts were categorised according to infection status (susceptible, *S*; infected, *I*; and recovered, *R*, where recovered hosts retained lifelong immunity), with susceptible hosts infected at the density-dependent rate *αδSI*. Increasing provisioning, tracked by the parameter *ρ*, reflects improved resource abundance and predictability, where *ρ *= 0 corresponds to no supplemental feeding and *ρ *= 1 reflects intensive provisioning. Provisioning here was assumed to be nutritionally complete, and parameter functional dependence on resources was assumed to be monotonic and saturating. If parameter *x* increased with provisioning, the functional form used was

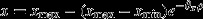

and if x decreased with provisioning, the relationship was described by

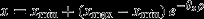

where *x*_min_ and *x*_max_ are the minimum and maximum values attained and *θ*_*x*_ describes the strength of the effect of provisioning. Through the shape parameter *θ*_*x*_, model parameters could scale with provisioning in forms that assume a weak but continuously increasing relationship to those assuming a strong, quickly saturating response.

Following expectations from the literature on the behavioural and physiological response of wildlife to provisioning, model parameters describing demographic rates (birth, *b*_*0*_ and mortality, *μ*), contact behavior (encounter rate, *α*), and immune defense (susceptibility, *δ* and tolerance, ν) were set to depend on *ρ*, with birth, contact, and tolerance increasing with provisioning and mortality and susceptibility decreasing with provisioning (Fig.4a). Since reduced susceptibility due to improved immune function would reduce transmission rates and therefore counter other changes that could increase pathogen spread (larger host population size, increased contact rates), the net effect of this interaction on infection dynamics was examined by covarying the strength of the responses of both susceptibility and tolerance to provisioning (*θ*_*δ*_ = *θ*_*υ*_ = *θ*_*δυ*_; an increasingly saturating effect is shown through line width in Fig.4b). The net effects of provisioning on pathogen invasion and outbreak capacity were inferred from analytic derivation of *R*_0_ in the SIR system. Further details, model parameterisation, and long-term epidemiological consequences of provisioning (equilibrium prevalence) are provided by Becker & Hall (2014). Ordinary differential equations of the SIR model and the analytic expression for *R*_0_ are given in the Supporting Information.

Simulations generated a range of *R*_0_ outcomes based on specific effects of provisioning (Fig.4c). When provisioning affected host demography and contact behaviour but not susceptibility and tolerance (dashed line, Fig.4c), this resulted in a dramatic increase in *R*_0_. However, this effect was modulated or even reversed when provisioning increased host immune response (increasing line width, Fig.4c). In this case of strong effects on host susceptibility and tolerance, *R*_0_ was minimised below the invasion threshold (*R*_0_ = 1) at intermediate levels of provisioning, indicating that provisioning can terminate epidemics. Hence depending on the response of host immune defence and the magnitude of provisioning, anthropogenic resources might result in explosive outbreaks and enhance pathogen fitness or could minimise prevalence and allow for pathogen extinction.

Some recent studies have extended models of local-scale dynamics to account for spatial heterogeneity in resource provisioning. As one example, a spatial model of Hendra virus dynamics in flying foxes examined how the combination of host aggregation around urban resources and resulting sedentary behaviour and loss of connectivity influenced viral invasion and persistence (Plowright *et al*. 2011). In particular, decreasing connectivity associated with urban areas increased epidemic size by increasing the duration of time between viral introductions, allowing subpopulations to recruit more susceptibles in the absence of infection and permitting the local loss of herd immunity. When decreased connectivity was modelled alongside urban aggregation, simulations produced the largest viral outbreaks in urban bat colonies, likely increasing the risk of spillover infections to other species (Plowright *et al*. 2011).

Modelling work on resource-driven infection dynamics to date has generally focused on microparasites, ignoring the heterogeneities in infection intensity and external transmission stages that characterise most macroparasites (Anderson & May 1978; Dobson & Hudson 1992). Importantly, expressions for *R*_0_ in macroparasite models depend on several parameters not represented in microparasite models, including the rate of production of free-living stages by adult worms, host uptake of infectious stages from the environment, and the mortality rate of adult parasites within their hosts, all of which could be influenced by provisioned resources. For example, well-fed hosts might provide better environments for macroparasite reproduction and survival, translating into greater *R*_0_ (Seppälä *et al*. 2008). Shifts away from natural food sources could also reduce exposure to infective stages, with the opposite effect of lowering *R*_0_ (Aponte *et al*. 2014). Future work that builds these resource-dependent relationships into macroparasite models will offer important advances for understanding divergent infection outcomes of resource provisioning.

### Predicting the effects of provisioning on *R*_0_ of a microparasite

We integrated modelling and empirical work by building the best-supported relationships from our meta-analysis into a mathematical model to examine effects on pathogen invasion (*R*_0_). Following the framework of Becker & Hall (2014), we set parameters for a susceptible–infected–recovered model to depend on resource levels (Box 1 and Supporting Information) and examined two different transmission scenarios (close contact versus dietary exposure). Because our meta-analysis suggests that anthropogenic provisioning might generally increase host susceptibility to infection (Fig.2b), we assume an increasing per-contact probability of infection (*δ*) with provisioning. We also assume that host tolerance increases with provisioning, by modelling the disease-induced mortality rate (*ν*) as a negative function of resources. Together, these two processes elevate *R*_0_. For pathogens transmitted by close contact, our analysis supported greater aggregation of hosts around resources, which likely increases contact rates (*α*). For pathogens transmitted through dietary exposure, studies indicated that provisioned diets could bypass parasite infectious stages (especially intermediate hosts). Finally, although our analysis found no significant support for resource-altered demography in driving infection, over half of the studies examined here found that provisioning affected demographic variables. We therefore follow Becker & Hall (2014) in assuming host birth (*b*_0_) increases and background mortality (*μ*) decreases with provisioning, but vary the strength of how these parameters respond to provisioning (as described in Box 1). Thus, our revised modelling framework includes increased host susceptibility and tolerance to infection alongside a range of weak to strong positive effects on host fecundity and survival. To account for different scenarios in which provisioning could (1) increase host contact or (2) decrease dietary exposure, we perform two simulations that capture these processes separately (through positive versus negative associations between provisioning and host exposure, *α*).

**Figure 5:**
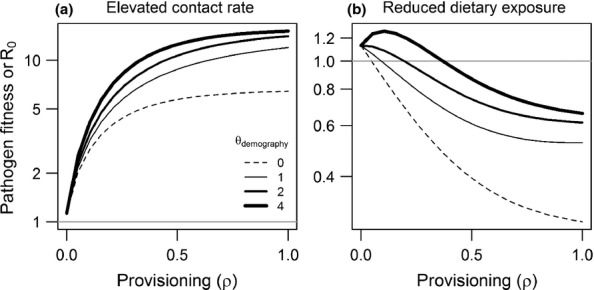
Meta-analysis-guided re-assessment of provisioning effects on pathogen invasion via mathematical models. Simulations examine net effects of resource-mediated processes on *R*_0_ by considering two independent behavioural mechanisms supported by our analysis, in which provisioning either elevates contact rates (a) or decreases dietary exposure to pathogens (b). Along with incorporating the above effects and those of resource-altered resistance and tolerance, the model includes potential influence of resource-altered demography, where line width indicates how strongly birth and mortality parameters respond to provisioning (shown in the legend). Simulations follow the parameterisation given in Becker & Hall (2014), and the analytic expression for *R*_0_ is provided in the Supplemental Material.

Our new simulations show that when provisioning increases host contact rates, the net outcome is an increase in *R*_0_ (Fig.5a). Even when host birth and background mortality remain unchanged, greater provisioning elevates *R*_0_ far above baseline levels due to the combined effects of increased contact, higher susceptibility, and improved host tolerance. When provisioning increases host birth rates and survival, we observe an even stronger increase in *R*_0_ similar to that found by Becker & Hall (2014). These interactive processes would predict a net positive influence of provisioning on pathogen fitness, consistent with some studies in our analysis but counter to the average trend (Fig.2a).

Importantly, modifying the model to assume that provisioning reduces dietary exposure to pathogens predicts different outcomes for *R*_0_ (Fig.5b). Under this scenario, when host fecundity and lifespan are unaffected by provisioning, reduced dietary exposure drives the pathogen to extinction, despite greater host susceptibility and tolerance to infection. If host survival and fecundity increase with provisioning, the pathogen can invade and persist at low to moderate resource levels. At high levels of provisioning, resource-altered dietary exposure dominates the overall effect on *R*_0_, driving the pathogen below the invasion threshold. This model prediction might explain cases in our analysis where supplemental resources decreased infection measures or had no net effect. The sensitivity of our model both to pathogen exposure routes and to demographic processes further highlights the need for detailed empirical studies of underlying mechanisms to understand outcomes for different wildlife–pathogen interactions.

## Management implications, future directions, and concluding remarks

Given the diverse responses of wildlife behaviour, immunity, and demography to dietary provisioning, and the potential for these changes to alter pathogen transmission within and between species, an important question is whether and how to manage pathogen risks to humans and wildlife arising from provisioning. Our analyses suggest that focusing on specific food sources and pathogen groups could improve disease management, as these together explained substantial variation in infection outcomes (Table2). In cases where microparasites are spread through close contact, solutions might involve spacing apart feeding stations to limit host aggregation, maintaining natural food sources, or preventing access to anthropogenic food altogether. As one example of this approach, in Uganda, better management of livestock grazing and encouraging the conservation of natural forest habitats have been proposed to mitigate enteric bacterial transmission between humans, domestic animals, and wild primates, the latter of which frequently forage in agricultural fields (Goldberg *et al*. 2008). For helminths or environmentally transmitted microparasites, solutions might involve periodic rotation or cleaning of feeding stations to limit the build-up of persistent infectious stages (Palmer & Whipple 2006). When anthropogenic resources are found to lower host immune defences, food could be fortified to make wildlife diets more nutritionally balanced (Knapp *et al*. 2013). Moreover, wildlife managers could use supplemented food sources to distribute vaccines or treatment to wildlife, taking advantage of oral bait vaccines such as those used for rabies and bovine tuberculosis (Boulanger *et al*. 2008; Gortazar *et al*. 2011). Finally, public outreach to promote awareness of how supplemental feeding affects the spread of wildlife pathogens or poses risks for human exposures might reduce transmission opportunities and limit human–wildlife contacts that allow pathogens to move in either direction.

Understanding how wildlife–pathogen dynamics respond to provisioning offers exciting challenges for new work (Box 2). Future studies could focus on systems where supplemental feeding is already known to affect host population dynamics or community interactions, but for which direct effects of feeding on pathogen transmission have not yet been quantified. For example, despite the popularity of recreational bird feeding (Robb *et al*. 2008), our review identified only four studies in which avian disease was explicitly quantified in the context of supplemental food (Fig. S2). Work in these tractable systems would benefit from longitudinal and experimental approaches, especially necessary to test how provisioning affects host immune defences, demography, and rates of recovery and pathogen shedding. To this end, researchers might capitalise on the human–wildlife connection inherent in provisioning by involving the public through citizen science projects and engaging with civic and recreational organisations during the design of wildlife surveillance programs. Similarly, collaboration with sociologists, anthropologists, and geographers can elucidate behavioural and socioeconomic drivers of provisioning and quantify human–wildlife interactions around these resources to better understand risks of human exposures and guide control strategies (Janes *et al*. 2012).

**Box 2 Outstanding needs for future work at the interface of provisioning and wildlife–pathogen dynamics**

1. Move beyond associational field studies
  - More intensive longitudinal and spatial monitoring of provisioned populations are needed to capture different resource levels and seasonality in responses.
  - Need to better quantify underlying mechanisms (immune defence, contact behaviour, dietary avoidance, birth and death rates) in the field.
  - Experimental manipulations of food sources and pathogen infection (i.e. pathogen removal studies) are needed to move beyond correlational outcomes.
2. Examine within-host responses to resources
  - Experimental studies of ecologically relevant field systems could test how diet quality (protein and energy content) and quantity (abundance and distribution) influence immune defence (including innate and adaptive immune pathways) and susceptibility and tolerance to specific pathogens.
  - Field and experimental studies are needed to ask how dietary shifts through provisioning affect the host microbiome and the resulting consequences for host condition and individual susceptibility to infection.
3. Develop new modelling approaches
  - Macroparasite models are needed to develop a mechanistic understanding of how environmentally transmitted and complex life cycle parasites respond to novel resources.
  - Spatial models that account for effects of resource heterogeneity on local dynamics and movement connectivity will be essential for understanding the persistence and spatial spread of infection.
4. Community context and host–pathogen evolution
  - Multihost modelling frameworks could explore how differential species contributions to parasite fitness are altered by the presence of novel resources, including broader potential for dilution or amplification effects on disease risk.
  - Predator–prey–pathogen models could ask how provisioned resources for predators and prey alter dynamical interactions.
  - Field and modelling studies are needed to understand whether and how supplemental feeding could influence the evolution of pathogen virulence and host resistance/tolerance to infection.

Our analysis demonstrates the utility of mathematical modelling to predict how anthropogenic resources affect host–pathogen dynamics. Such theoretical approaches have mainly focused on microparasite transmission, and a need remains to develop macroparasite models that capture effects of food provisioning. Building on established frameworks for helminth dynamics (Anderson & May 1978), future models could examine how resource dependence influences adult parasite survival and egg production, parasite impacts on host survival and fecundity, and parasite encounter rates through host foraging (Dobson & Hudson 1992). Another important step for mathematical models is the development of spatial frameworks that capture local- and regional-scale heterogeneity in provisioning and allow for host movement between provisioned and unprovisioned patches (Hanski 1999; Plowright *et al*. 2011). Finally, future modelling studies can borrow from community ecology to understand more complex interactions between hosts, pathogens, and resources, including multiple host or pathogen species or predators and their prey.

From an evolutionary perspective, an exciting avenue for future work is to ask how provisioning might affect host resistance and pathogen virulence evolution. Increased opportunities for pathogen transmission are expected to favour the evolution of more virulent pathogen strains (Levin 1996; De Roode *et al*. 2008), but empirical data to test this prediction in the context of transmission opportunities arising from provisioning are lacking. From a different perspective, our analysis suggests that anthropogenic resources can in some cases allow wildlife to better tolerate infection. This association is corroborated by laboratory evidence demonstrating improved nutrition prolongs the survival of infected animals and increases the duration of pathogen shedding (Brown *et al*. 2000; Vale *et al*. 2013). More tolerant hosts could further select for faster-replicating pathogen strains that cause greater virulence (Vale *et al*. 2011). Thus, although resource-improved condition could reduce disease-induced mortality in the short term, provisioning could favour the evolution of more harmful pathogen strains in the longer term (Miller *et al*. 2006). Evolutionary models exploring the impact of improved tolerance within the context of other immune, behavioural, and demographic effects are needed to predict the long-term consequences of provisioning for wildlife and human health.

As human populations expand, natural habitats and food sources for many wildlife species will continue to be replaced by human-dominated landscapes and anthropogenic resources. These changes will have profound effects on the spatial and temporal distribution of wildlife and on their interactions with parasites and pathogens. Our review underscores the need to better understand how food resources affect wildlife physiology and behaviour and how changes at individual and local scales alter landscape-level pathogen dynamics. Our synthesis of evidence to date highlights the fact that provisioning effects on infection depend crucially on details of the host–pathogen interaction; however, some generalities nevertheless arise based on pathogen type, transmission mode, sources of novel food, and the relative impacts on host behaviour and immunity. Future integration of long-term field studies, experimental approaches, and mathematical models of provisioning are needed to define more robust mechanistic frameworks and to guide efforts to mitigate infection risks for wildlife, domesticated animals, and humans.
