# Supplementary material for: Linking anthropogenic resources to wildlife–pathogen dynamics: a review and meta-analysis
Source: Ecol Lett. 2015 Mar 21;18(5):483–95. doi: 10.1111/ele.12428 (PMC4403965; doi:10.1111/ele.12428)
Supplement: Supplementary file 1 [file ele0018-0483-sd1.docx]

**Linking anthropogenic resources to wildlife–pathogen dynamics: a review and meta-analysis: Supplementary Material**

Daniel J. Becker, Daniel G. Streicker, and Sonia Altizer

**Data collection procedure**

**Descriptive meta-analysis**

**Publication bias**

**Predictors of host–pathogen responses to anthropogenic resources**

**Sensitivity to studies with proximate measures of provisioning**

**Studies included in the meta-analysis**

**Model equations and derivation of R_0_**

**Works cited**

**Data collection procedure**

We here present additional detail on the collection and distribution of data included in our meta-analysis of the relationship between resource provisioning and infection outcomes in wildlife. Systematic searches were performed in Web of Science, Google Scholar, CAB Abstracts, and PubMed. Because the search interfaces are similar, we used the same strings of search terms in Web of Science, CAB Abstracts, and PubMed.

("anthropogenic food" OR "anthropogenic resource" OR provisioning OR provisioned OR "supplemental feed" OR "supplemental resource" OR "resource supplementation" OR "resource subsidies" OR "anthropogenic subsidies" OR "human-provided subsidies" OR "human-provided resource" OR "host resource")

AND

(disease* OR infect* OR pathogen* OR bacteria* OR parasite* OR virus* OR helminth* OR protozoa* OR epidemic* OR transmission*)

AND (wildlife OR wild)

The Web of Science search was restricted by research domain (SCIENCE TECHNOLOGY) and by research area (ENVIRONMENTAL SCIENCES ECOLOGY OR BIODIVERSITY CONSERVATION OR PATHOLOGY OR ZOOLOGY OR EVOLUTIONARY BIOLOGY OR IMMUNOLOGY OR HEMATOLOGY OR INFECTIOUS DISEASES OR MICROBIOLOGY OR VETERINARY SCIENCES OR PARASITOLOGY OR VIROLOGY).

Similarly, our Google Scholar search contained the following search string.

("anthropogenic food" OR provisioning OR "supplemental feed*" OR "supplemental resource*" OR "resource subsidies*" OR supplementation) AND (disease* OR infect* OR pathogen* OR transm* ) AND wildlife

We restricted searches from 2014 back to 1990, when the last comprehensive review of wildlife supplementation experiments was published (Boutin 1990).

Together these searches resulted in 2216 records. We then followed the PRIMSA documentation procedure to record the results and exclusion process for the systematic search (Moher *et al.* 2009). Each title and abstract was first screened for duplication and basic criteria for inclusion in our analysis of wildlife provisioning. The majority of records were initially removed as they consisted of human studies, toxicological studies, review papers, theoretical studies, and antibiotic and probiotic supplement experiments. Furthermore, studies were retained during this phase only if they examined effects of provisioning in wild or free-ranging populations. Together these screening criteria narrowed our search to 231 articles that were assessed for eligibility in the full-text.

This in-depth process excluded an additional 87 studies owing either to the above criteria or to a lack of comparison between provisioned and unprovisioned groups. Therefore we have included 144 studies in our overall qualitative synthesis, of which 23 (16%) recorded measures of disease and are included in the meta-analysis. The complete inclusion process is documented below in flow diagram form (Figure S1).

Records identified through searching Google Scholar and Web of Science (n = 1857)

## Screening

## Included

## Eligibility

## Identification

Additional records identified through other sources (CAB Abstracts, PubMed) (n = 359)

Records after duplicates removed
(n = 2016)

Records screened
(n = 2016)

Records excluded
(n = 1785)

Full-text articles assessed for eligibility
(n = 231)

Full-text articles excluded for not comparing provisioned and unprovisioned wildlife (n = 87)

Studies included in qualitative synthesis
(n = 144)

Studies included in quantitative synthesis (meta-analysis)
(n = 23)

Figure S1. PRIMSA diagram documenting the data collection and inclusion process.

For each line of data in the 23 studies documenting infection differences between provisioned and unprovisioned wildlife, we recorded host and pathogen type, pathogen transmission mode, if provisioning was accidental or intentional, and the reported source of provisioning. Host type was recorded as phylogenetic class, and pathogen type was classified as bacterium, virus, fungus, protozoan, ectoparasite, or helminth. We determined transmission mode by matching our parasite species or genus records to those of the Global Mammal Parasite Database (GMPD), the most comprehensive collection of published records of parasitic organisms from free-living mammals (Nunn & Altizer 2005). Transmission mode was defined using the GMPD classifications of close contact, non-close contact, vector-borne, and complex life cycle (following Pedersen et al. 2005). However, as 21% (n = 28) of our data were from non-mammals (23 lines Aves, 2 lines Chondrichthyes, 1 line Reptilia), we verified transmission mode from a random sample (n = 9 lines, 30%) of these other host–pathogen interactions using Acha & Szyfres (2003). When transmission mode for a particular pathogen species was not included in this reference, we matched transmission to that reported for the genus or family. If transmission mode was not listed at this level, we matched GMPD classifications to the mode of transmission listed in the study (e.g., Aegidae parasites; Semeniuk & Rothley 2008). We found 100% identify between the GMPD and these references for our sample.

For each study, we also recorded the reported source of provisioned resources, and the majority of studies directly included provisioning as categorical (e.g., provisioned habitat type or population) or continuous (e.g., quantity of supplemental feed) variables. The remaining studies indirectly incorporated provisioning through explicit mention of anthropogenic resources in habitat type (e.g., urban environments having more abundant food compared to rural counterparts; Cypher & Frost 1999; Hill *et al.* 2008) or through proximal measures (e.g., urbanization score as an indirect measure of bird feeder abundance; Bradley *et al.* 2008). We then classified provisioning sources into categories of agriculture (crop fields), urban waste (landfills, household trash), management (feeding grounds, bait piles), and recreation (tourism, bird feeders). Studies directly including provisioning represented 83% (n = 19) of our dataset, while proximal measures were used in 17% (n = 4) of included studies (Table S1).

Table S1: Mode of inclusion of anthropogenic resources in studies of host–pathogen interactions and classification of provisioning type and wildlife food source.

| **study** | **resource inclusion** | **type of provisioning** | **reported source** | **category of resource** |
| --- | --- | --- | --- | --- |
| Aponte *et al.* 2014 | direct | accidental | urban waste | urban waste |
| Blanco *et al.* 2011 | direct | intentional | management | management |
| Bradley *et al.* 2008 | proxy | intentional | bird feeders | recreation |
| Cypher & Frost 1999 | proxy | accidental | urban waste | urban waste |
| Hegglin *et al.* 2007 | direct | accidental | urban waste | urban waste |
| Hill *et al.* 2008 | proxy | accidental | urban waste | urban waste |
| Hines *et al.* 2007 | direct | intentional | feed grounds | management |
| Knapp *et al.* 2013 | direct | intentional | tourism | recreation |
| Lane *et al.* 2011 | direct | intentional | tourism | recreation |
| Lonsdorf *et al.* 2011 | direct | intentional | banana feeding | management |
| Luong *et al.* 2014 | direct | intentional | bait piles | management |
| Miller *et al.* 2003 | direct | intentional | feed grounds | management |
| Monello & Gompper 2010 | direct | Intentional | bait piles | management |
| Monello & Gompper 2011 | direct | intentional | bait piles | management |
| Navarro-Gonzalez *et al.* 2013 | direct | intentional | hunting estate | management |
| Page *et al.* 2008 | direct | accidental | urban waste | urban waste |
| Robardet *et al.* 2008 | direct | accidental | urban waste | urban waste |
| Semeniuk & Rothley 2008 | direct | intentional | tourism | recreation |
| Vicente *et al.* 2007a | direct | intentional | feed grounds | management |
| Vicente *et al.* 2007b | direct | intentional | feed grounds | management |
| Wenz-Mucke *et al.* 2013 | direct | accidental | picnic raiding | recreation |
| Wright & Gompper 2005 | direct | intentional | bait piles | management |
| Zylberberg *et al.* 2013 | proxy | accidental | crop fields | agriculture |

**Descriptive meta-analysis**

Although the study of provisioning impacts on wildlife populations has increased over time ($F_{1,18}$ = 28.14, $p$ < 0.001), such a trend has not followed in regards to the proportion of studies quantifying disease outcomes ($F_{1,18}$= 3.26, $p$ = 0.09). Out of 144 studies of provisioning identified since 1990, only 23 quantified infection outcomes between provisioned and unprovisioned populations (Fig. S2a). For studies quantifying disease, we found a strong tendency toward studying mammals, which constituted 79% (n = 104) of our dataset (Fig. S2b). Protozoa and helminths were the most common pathogens studied (69%, n = 91), with the remaining host–pathogen interactions represented by viruses (12%, n = 16), bacteria (10%, n = 13), ectoparasites (8%, n = 11), and fungi (1%, n = 1). The majority of studies (65%, n = 15) observed relationships between intentional forms of provisioning (management practices, recreation) and disease outcomes, with 8 studies examining accidental provisioning (Fig. S2c).

In terms of quantifying potential mechanisms driving effects of provisioning on disease, 52% (n = 12) of studies quantified host condition or immune defense (Fig. S3a). Of the measures of condition or immune function quantified, calculation of body condition was the most common variable recorded. 43% (n = 10) of studies quantified a behavioral response of hosts, in which aggregated group size around anthropogenic resources and measures of dietary breadth were the most common measures. Additionally, 26% (n = 6) of studies in our dataset quantified demographic factors in response to provisioning, with host density and population size being measured. When recording the response of these measures to provisioning, we observed a roughly even division between condition and immune measures increasing, decreasing, or showing no effect (Fig. S3b). In contrast measures of behavioral change only showed positive or negative effects, with positive denoting increases in aggregation or contact while negative denotes simplification of host diet. Demographic variables tended to either increase or not be affected by anthropogenic resources (Fig. S3b).

Our analysis of this dataset used random (REM) and mixed-effects (MEM) models to infer the average effect of provisioning. The REM estimates the average true effect ($\mu$) and total heterogeneity among true effects ($\tau^{2}$), while the MEM includes study-level variables to explain variation in outcomes (Gurevitch & Hedges 1999). We estimated $\mu$ and $\tau^{2}$ using maximum likelihood with sample size incorporated into the calculation of weights (Raudenbush 2009) and test against the null hypotheses of $\mu$=0 and $\tau^{2}$=0 using the Wald and Cochran’s $Q$ test, respectively (Higgins & Thompson 2002). We then used MEM to explain variation in infection according to covariates related to pathogen traits and host foraging ecology. We identified the strongest predictors of infection through a model simplification procedure in which the full MEM included pathogen type, transmission mode, host taxonomy, and provisioning type and source. Model simplification used backward removal of the least significant variable using Wald-type chi-square tests followed by nested likelihood ratio tests (Van Houwelingen *et al.* 2002).

**
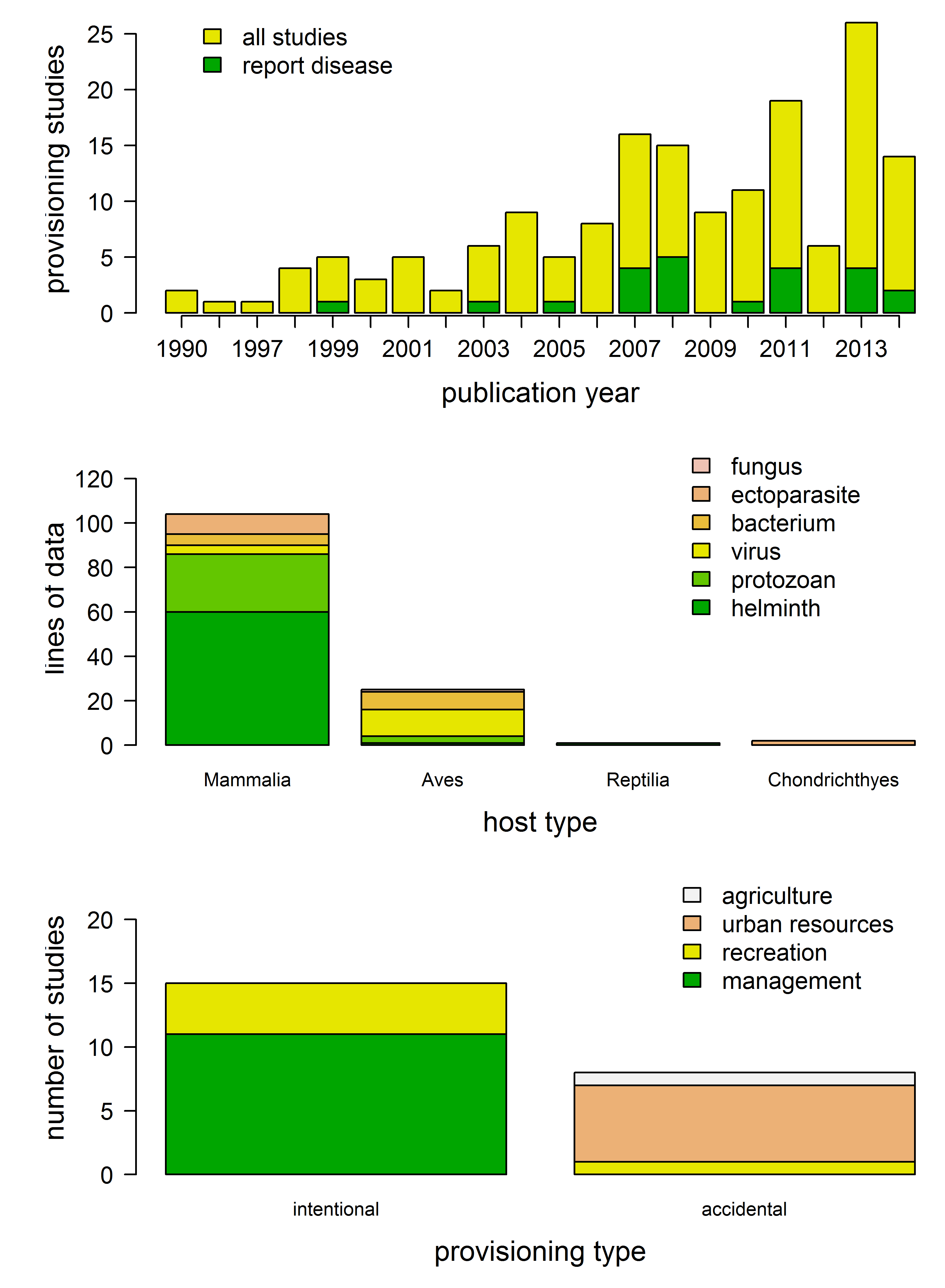
**

Figure S2. Distribution of meta-analysis data by (a) publication year, (b) host and pathogen type, and (c) provisioning intentionality and anthropogenic food source.

**
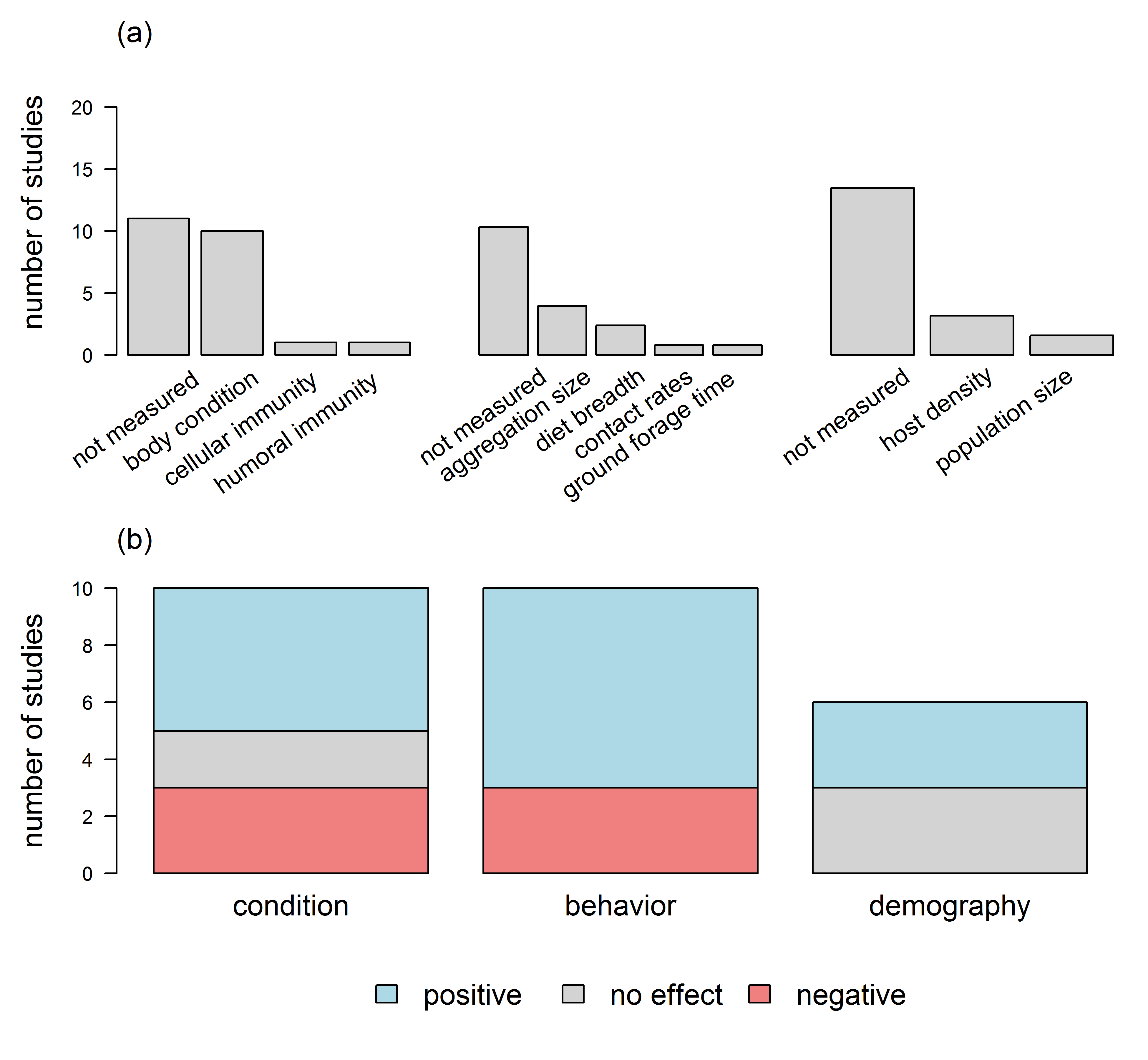
**

Figure S3. Distribution of (a) mechanism variables measured and (b) the directional effect of provisioning on each variable subset.

**Publication bias**

In addition to the main aims of our analysis, we assessed publication bias in our dataset using funnel plots displaying standardized effect sizes against their corresponding standard errors. A symmetrical plot indicates no publication bias, as effects from studies with high precision will remain close to the estimated true effect, while effects from studies with large standard errors should be spread equally on both sides of the mean (Egger *et al.* 1997; Sterne & Egger 2001). We evaluated funnel plot asymmetry for the REM using the rank correlation test, which tests for a relationship between observed effect sizes and standard errors (Begg and Mazumdar, 1994; Sterne and Egger, 2005). We further explored publication bias using the trim and fill method (Duval & Tweedie 2000), a non-parametric rank-based technique that estimates the number of studies missing from analysis due to suppression of extreme and non-significant observations and tests if addition of these results would affect the estimation of the mean true effect.

Using the REM Egger test, we did not detect a relationship between effect size and standard error ($z$ = 1.83, $p$ = 0.07), suggesting a lack of publication bias in the study of provisioning and wildlife disease (Fig. S4a). However, trim and fill analyses using the L0^+^ estimator suggested 45 lines of data demonstrating effects less than the estimated true effect ($\mu$ = 0.096) were missing from analysis due to suppression of extreme or non-significant results. Incorporating these missing lines of data shifted the confidence interval of the estimated true effect to cross zero (Fig. S4b), negating the observed positive relationship between provisioning and disease outcomes observed in our main analysis ($\mu$ = –0.06, $z$ = –1.80, $p$ = 0.07).


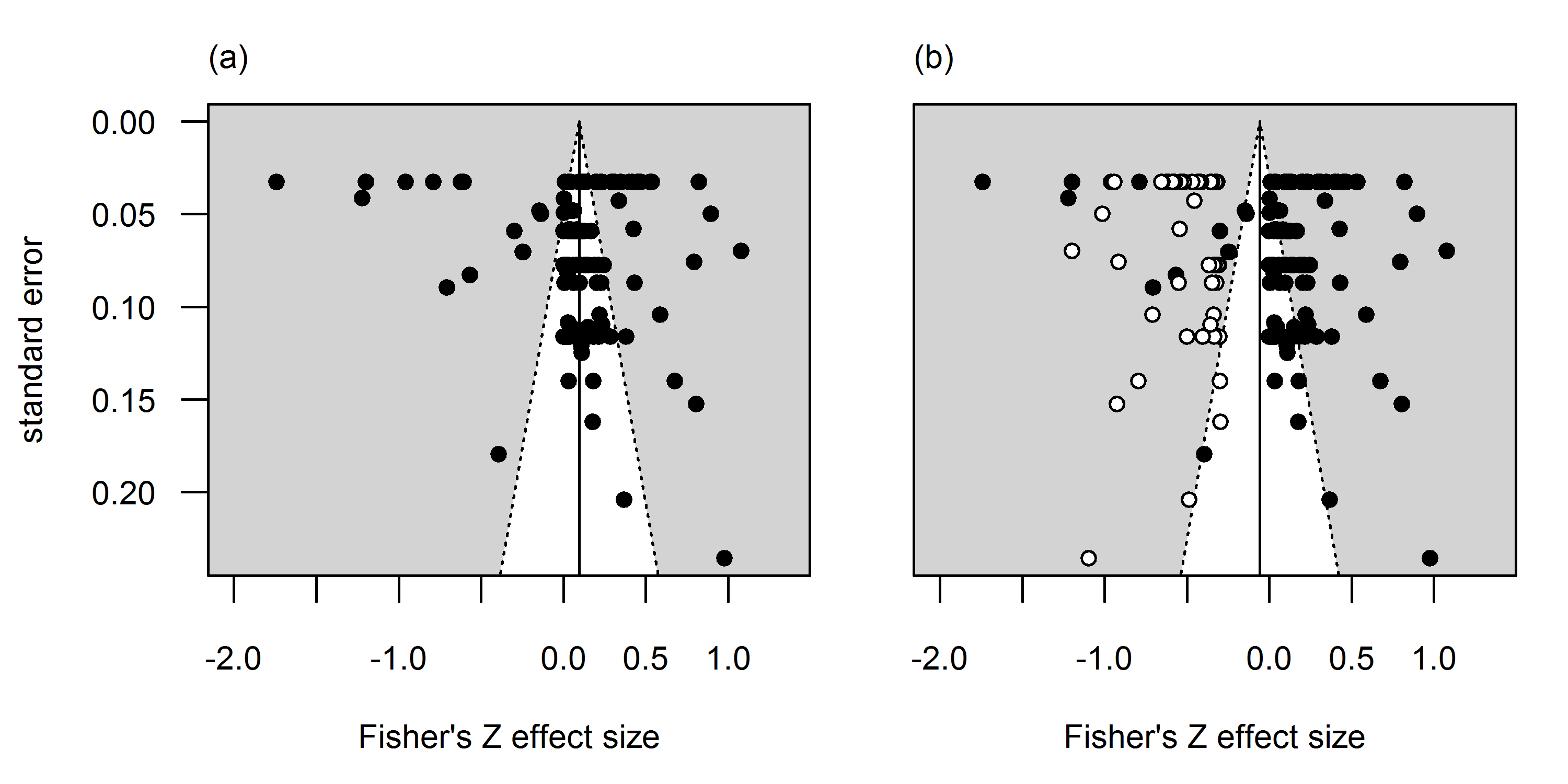


Figure S4. Funnel plots illustrating (a) the relationship between effect size and standard error and (b) the effect of correcting funnel plot asymmetry through trim and fill analysis on the estimated true effect of provisioning on disease outcomes.

**Predictors of host–pathogen responses to anthropogenic resources**

MEM analyses demonstrated significant effects of all individual covariates on infection outcomes in provisioned populations, although model comparison identified provisioning source and pathogen type as individually explaining the most variation (Table S2).

Table S2: Univariate MEM analyses ranked by AIC

| predictor variables | *τ^2^* | *Q_M_* | *Q_M_* p-value | ΔAIC | *R^2^* (%) |
| --- | --- | --- | --- | --- | --- |
| ~ provisioning source | 0.12 | 19.71 | 0.001 | 0 | 7.58 |
| ~ provisioning type | 0.13 | 12.18 | 0.002 | 2 | 2.51 |
| ~ pathogen type | 0.13 | 18.44 | 0.005 | 5 | 6.77 |
| ~ host taxonomy | 0.13 | 12.44 | 0.014 | 6 | 2.59 |
| ~ transmission mode | 0.13 | 9.55 | 0.049 | 9 | 0.61 |

We here visualize the influence of the other three predictors (Fig. S5). Host taxonomy and provisioning source explained roughly equal amounts of variation in infection outcomes, with intentional forms of provisioning and all classes of hosts associated with increased disease. Transmission mode explained the least amount of variation.

Figure S5. Remaining predictors of heterogeneity in infection outcomes of resource provisioning, ranked by AIC. Diamonds with 95% confidence intervals show the estimated mean effect size for each level of each factor.

**Sensitivity to studies with proximate measures of provisioning**

Our main tests used studies that directly included provisioning in their analyses alongside studies that used more proximal measures of anthropogenic resources. Because infection outcomes in these proximal studies could be affected by various other factors of urbanized environments, we here present sensitivity analyses for a reduced dataset only containing studies where inclusion of provisioning was direct. Importantly, our results vary very little when proximal studies were excluded from analysis (Table S3). As differences between analyses were minimal, we justify inclusion of proximal studies in the main text to optimize statistical power.

We first ran REM and publication bias analyses only for studies directly including provisioning as a variable of interest (Table S3). As in the REM analysis of our full dataset, after accounting for missing lines of data ($k$ = 42) due to suppression of extreme or non-significant results, there was no net effect of provisioning on infection for the reduced dataset ($\mu$ = –0.06, $p$ = 0.06). We similarly did not detect a relationship between effect sizes and standard errors when excluding proximal studies ($z$ = 1.79, $p$ = 0.07) using the Egger test.

MEM analysis of this reduced dataset also differed little (Table S4). Stepwise model simplification again identified pathogen type and source as together explaining the most variation in infection outcomes (20.81%) in comparison to the base REM ($LRT$ = 28.7, $df$ = 2, $p$ < 0.001). As in the full analysis, the reduced analysis indicated that management- and recreation-based provisioning sources promote increased infection outcomes, whereas foraging on urban waste was associated with reduced disease. Similarly, the reduced dataset also found that the transmission of bacteria, viruses, and helminths are expected to increase in response to provisioning. Lastly, analyses of the reduced dataset likewise supported behavioral and immunological mechanisms by which provisioning affects infection outcomes, including greater aggregation, dietary simplification, and improved tolerance predicting higher infection rates.

Table S3: Results of REM and publication bias tests for full and reduce dataset

|  | **records** | **μ** | **p** | **Q_E_** | **p** | **Egger’s test z** | **p** | **missing lines** | **bias-corrected μ** | **p** |
| --- | --- | --- | --- | --- | --- | --- | --- | --- | --- | --- |
| full | 132 | 0.095 | 0.004 | 11274 | < 0.001 | 1.83 | 0.07 | 45 | -0.059 | 0.07 |
| reduced | 126 | 0.090 | 0.008 | 11206 | < 0.001 | 1.79 | 0.07 | 42 | -0.063 | 0.06 |

Table S4: Results of MEM analyses for full and reduce dataset

|  | **best model** | **R^2^** | **Q_M_ condition** | **p** | **Q_M_ behavior** | **p** | **Q_M_ demography** | **p** |
| --- | --- | --- | --- | --- | --- | --- | --- | --- |
| full | ~ pathogen + source | 17.85 | 24.84 | < 0.001 | 45.29 | < 0.001 | 3.59 | 0.17 |
| reduced | ~ pathogen + source | 20.81 | 20.48 | < 0.001 | 45.29 | < 0.001 | 3.59 | 0.17 |

**Studies included in the meta-analysis**

1. Aponte, V., Locke, S.A., Gentes, M.-L., Giroux, J.-F., Marcogliese, D.J., McLaughlin, D., *et al.* (2014). Effect of habitat use and diet on the gastrointestinal parasite community of an avian omnivore from an urbanized environment. *Canadian Journal of Zoology*, 92, 1–8.
2. Blanco, G., Lemus, J.A. & García-Montijano, M. (2011). When conservation management becomes contraindicated: impact of food supplementation on health of endangered wildlife. *Ecological Applications*, 21, 2469–2477.
3. Bradley, C.A., Gibbs, S.E.J. & Altizer, S. (2008). Urban land use predicts West Nile virus exposure in songbirds. *Ecol Appl*, 18, 1083–1092.
4. Cypher, B.L. & Frost, N. (1999). Condition of San Joaquin kit foxes in urban and exurban habitats. *J. Wildl. Manage.*, 63, 930–938.
5. Hegglin, D., Bontadina, F., Contesse, P., Gloor, S. & Deplazes, P. (2007). Plasticity of predation behaviour as a putative driving force for parasite life-cycle dynamics: the case of urban foxes and Echinococcus multilocularis tapeworm. *Functional Ecology*, 21, 552–560.
6. Hill, N.J., Deane, E.M. & Power, M.L. (2008). Prevalence and genetic characterization of Cryptosporidium isolates from common brushtail possums (Trichosurus vulpecula) adapted to urban settings. *Applied and environmental microbiology*, 74, 5549–5555.
7. Hines, A.M., Ezenwa, V.O., Cross, P. & Rogerson, J.D. (2007). Effects of supplemental feeding on gastrointestinal parasite infection  in elk (Cervus elaphus): Preliminary observations. *Vet. Parasitol.*, 148, 350–355.
8. Knapp, C.R., Hines, K.N., Zachariah, T.T., Perez-Heydrich, C., Iverson, J.B., Buckner, S.D., *et al.* (2013). Physiological effects of tourism and associated food provisioning in an endangered iguana. *Conserv Physiol*, 1, cot032.
9. Lane, K.E., Holley, C., Hollocher, H. & Fuentes, A. (2011). The anthropogenic environment lessens the intensity and prevalence of gastrointestinal parasites in Balinese long-tailed macaques (Macaca fascicularis). *Primates*, 52, 117–128.
10. Lonsdorf, E.V., Murray, C.M., Lonsdorf, E.V., Travis, D.A., Gilby, I.C., Chosy, J., *et al.* (2011). A Retrospective Analysis of Factors Correlated to Chimpanzee (Pan troglodytes schweinfurthii) Respiratory Health at Gombe National Park, Tanzania. *EcoHealth*, 8, 26–35.
11. Luong, L.T., Grear, D.A. & Hudson, P.J. (2014). Manipulation of host-resource dynamics impacts transmission of trophic parasites. *International Journal for Parasitology*.
12. Miller, R.A., Kaneene, J.B., Fitzgerald, S.D. & Schmitt, S.M. (2003). Evaluation of the influence of supplemental feeding of white-tailed deer (Odocoileus virginianus) on the prevalence of bovine tuberculosis in the Michigan wild deer population. *Journal of Wildlife Diseases*, 39, 84–95.
13. Monello, R.J. & Gompper, M.E. (2010). Differential effects of experimental increases in sociality on ectoparasites of free-ranging raccoons. *Journal of animal ecology*, 79, 602–609.
14. Monello, R.J. & Gompper, M.E. (2011). Effects of resource availability and social aggregation on the species richness of raccoon endoparasite infracommunities. *Oikos*, 120, 1427–1433.
15. Navarro-Gonzalez, N., Fernández-Llario, P., Pérez-Martín, J.E., Mentaberre, G., López-Martín, J.M., Lavín, S., *et al.* (2013). Supplemental feeding drives endoparasite infection in wild boar in Western Spain. *Veterinary parasitology*, 196, 114–123.
16. Page, L.K., Gehrt, S.D. & Robinson, N.P. (2008). Land-use effects on prevalence of raccoon roundworm (Baylisascaris procyonis). *Journal of wildlife diseases*, 44, 594–599.
17. Robardet, E., Giraudoux, P., Caillot, C., Boue, F., Cliquet, F., Augot, D., *et al.* (2008). Infection of foxes by Echinococcocus multilocularis in urban and  suburban areas of Nancy, France: Influence of feeding habits and  environment. *Parasite-J. Soc. Fr. Parasitol.*, 15, 77–85.
18. Semeniuk, C.A. & Rothley, K.D. (2008). Costs of group-living for a normally solitary forager: effects of provisioning tourism on southern stingrays Dasyatis americana. *MARINE ECOLOGY-PROGRESS SERIES-*, 357, 271.
19. Vicente, J., Höfle, U., Fernández-De-Mera, I.G. & Gortazar, C. (2007a). The importance of parasite life history and host density in predicting the impact of infections in red deer. *Oecologia*, 152, 655–664.
20. Vicente, J., Höfle, U., Garrido, J.M., Fernández-de-mera, I.G., Acevedo, P., Juste, R., *et al.* (2007b). Risk factors associated with the prevalence of tuberculosis-like lesions in fenced wild boar and red deer in south central Spain. *Veterinary Research*, 38, 451–464.
21. Wenz-Mücke, A., Sithithaworn, P., Petney, T.N. & Taraschewski, H. (2013). Human contact influences the foraging behaviour and parasite community in long-tailed macaques. *Parasitology*, 140, 709–718.
22. Wright, A.N. & Gompper, M.E. (2005). Altered parasite assemblages in raccoons in response to manipulated resource availability. *Oecologia*, 144, 148–156.
23. Zylberberg, M., Lee, K.A., Klasing, K.C. & Wikelski, M. (2013). Variation with Land Use of Immune Function and Prevalence of Avian Pox in Galapagos Finches. *Conserv. Biol.*, 27, 103–112.

**Model equations and derivation of R_0_**

The modeling framework outlined by Becker & Hall (2014) used simple compartmental models to describe the effect of provisioning on microparasite dynamics. The basic susceptible–infected–recovered system is described by three differential equations.

$${dS}/{dt}=\left( b_{0}-b_{1}\left( S+I+R \right) \right)(S+I+R)-\mu S-\alpha\delta SI$$

$${dI}/{dt}= \alpha\delta SI-\left( \mu+\nu+\gamma\right)I$$

$${dR}/{dt}= \gamma I-\mu R$$

Host demography is described by a natural mortality rate $\mu$ and a density-dependent birth rate $b_{0}-b_{1}N$, where $N$ is the population size and $b_{0}$ and $b_{1}$ are constants. Together these parameters give $K$, the host carrying capacity (${b_{0}-\mu}/{b_{1}}$). The framework assumes density-dependent pathogen transmission, but states the transmission parameter as the product of terms describing contact rate ($\alpha$) and probability of infection upon encounter ($\delta$). Pathogen clearance and disease-induced mortality occur at rates $\gamma$ and $\nu$ respectively.

For the SIR system, R_0_ is the product of the disease-free equilibrium host population size (i.e., carrying capacity), the transmission rate, and the infectious period (the inverse of the rate at which infected individuals leave the infectious class):

$$R_{0}=\frac{\alpha\delta K}{\mu+\upsilon+\gamma}$$

To derive the provisioning-dependent R_0_, parameters are set to depend on the functional forms stated in the main text (Box 1). Under provisioning, $\alpha$ and $b_{0}$ are increasing functions of the provisioning parameter $\rho$, whereas $\delta$, $\upsilon$, and $\mu$ are decreasing functions. R_0_ is then attained by substituting expressions for the resource-dependent parameters into the above equation (Otto & Day 2007; Becker & Hall 2014).

For our empirically revised modeling framework of microparasite transmission, we follow this same procedure for the analytic derivation of R_0_. However, unlike in Becker & Hall (2014), following our meta-analysis results we here set $\delta$ to increase with provisioning. Additionally, in the simulation examining the influence of reduced dietary exposure, we further alter this framework by changing $\alpha$ to decrease with provisioning. Parameter values are the same as those used in Becker & Hall (2014), although $\theta_{x}$ for resource-dependent parameters is here set equal for behavioral and immunological mechanisms.

**Works cited**

1. Acha, P.N. & Szyfres, B. (2003). *Zoonoses and Communicable Diseases Common to Man and Animals: Parasitic Zoonoses*. Pan American Health Org.

2. Becker, D.J. & Hall, R.J. (2014). Too much of a good thing: resource provisioning alters infectious disease dynamics in wildlife. *Biol. Lett.*, 10, 20140309.

3. Begg, C.B. & Mazumdar, M. (1994). Operating characteristics of a rank correlation test for publication bias. *Biometrics*, 1088–1101.

4. Boutin, S. (1990). Food supplementation experiments with terrestrial vertebrates: patterns, problems, and the future. *Can. J. Zool.*, 68, 203–220.

5. Bradley, C.A., Gibbs, S.E.J. & Altizer, S. (2008). Urban land use predicts West Nile virus exposure in songbirds. *Ecol. Appl. Publ. Ecol. Soc. Am.*, 18, 1083–1092.

6. Cypher, B.L. & Frost, N. (1999). Condition of San Joaquin kit foxes in urban and exurban habitats. *J. Wildl. Manag.*, 63, 930–938.

7. Duval, S. & Tweedie, R. (2000). A nonparametric “trim and fill” method of accounting for publication bias in meta-analysis. *J. Am. Stat. Assoc.*, 95, 89–98.

8. Egger, M., Smith, G.D., Schneider, M. & Minder, C. (1997). Bias in meta-analysis detected by a simple, graphical test. *Bmj*, 315, 629–634.

9. Gurevitch, J. & Hedges, L.V. (1999). Statistical issues in ecological meta-analyses. *Ecology*, 80, 1142–1149.

10. Higgins, J.P.T. & Thompson, S.G. (2002). Quantifying heterogeneity in a meta-analysis. *Stat. Med.*, 21, 1539–1558.

11. Hill, N.J., Deane, E.M. & Power, M.L. (2008). Prevalence and genetic characterization of Cryptosporidium isolates from common brushtail possums (Trichosurus vulpecula) adapted to urban settings. *Appl. Environ. Microbiol.*, 74, 5549–5555.

12. Van Houwelingen, H.C., Arends, L.R. & Stijnen, T. (2002). Advanced methods in meta-analysis: multivariate approach and meta-regression. *Stat. Med.*, 21, 589–624.

13. Moher, D., Liberati, A., Tetzlaff, J., Altman, D.G. & The PRISMA Group. (2009). Preferred Reporting Items for Systematic Reviews and Meta-Analyses: The PRISMA Statement. *PLoS Med*, 6, e1000097.

14. Nunn, C.L. & Altizer, S.M. (2005). The global mammal parasite database: an online resource for infectious disease records in wild primates. *Evol. Anthropol. Issues News Rev.*, 14, 1–2.

15. Otto, S.P. & Day, T. (2007). *A biologist’s guide to mathematical modeling in ecology and evolution*. Princeton University Press.

16. Pedersen, A.B., Altizer, S., Poss, M., Cunningham, A.A. & Nunn, C.L. (2005). Patterns of host specificity and transmission among parasites of wild primates. *Int. J. Parasitol.*, 35, 647–657.

17. Raudenbush, S.W. (2009). Analyzing effect sizes: Random-effects models. *Handb. Res. Synth. Meta-Anal.*, 2, 295–316.

18. Semeniuk, C.A. & Rothley, K.D. (2008). Costs of group-living for a normally solitary forager: effects of provisioning tourism on southern stingrays Dasyatis americana. *Mar. Ecol.-Prog. Ser.-*, 357, 271.

19. Sterne, J.A. & Egger, M. (2001). Funnel plots for detecting bias in meta-analysis: guidelines on choice of axis. *J. Clin. Epidemiol.*, 54, 1046–1055.
